# Risk and Protective Factors Associated with Custodial Grandparents’ Psychological Distress in COVID-19

**DOI:** 10.1093/geroni/igaa057.3495

**Published:** 2020-12-16

**Authors:** Yanfeng Xu, Merav Jedwab, Qi Wu, Sue Levkoff, Ling Xu

**Affiliations:** 1 University of South Carolina, Columbia, South Carolina, United States; 2 Hadassah Academic College, Jerusalem Israel, Israel; 3 Arizona State University, Phoenix, Arizona, United States; 4 The University of Texas at Arlington, Arlington, Texas, United States

## Abstract

The fear and anxiety of COVID-19 and its related policy measures have increased individuals’ psychological distress. The objective of this study was to examine relationships between material hardship, parenting stress, social support, and resilience and custodial grandparents’ psychological distress during the COVID-19 pandemic and further investigate the moderating role of kinship license status. A cross-sectional survey was administered to collect data from custodial grandparents (N = 362) in the United States. T-tests, chi-square tests, and logistic regression models were conducted using STATA 15.0. Results indicated that material hardship (OR = 1.77, p < 0.001) was associated with higher odds of psychological distress, whereas custodial grandparents’ resilience (OR = 0.08, p < 0.001) and social support (OR = 0.39, p < 0.001) were associated with lower odds of experiencing psychological distress. Increased parenting stress in COVID-19 was not significantly associated with psychological distress. Kinship license status moderated the relationships between social support (OR = 0.23, p < 0.05), resilience (OR = 5.06, p < 0.05) and psychological distress. To address custodial grandparents’ psychological distress, more allocated emergency funds and tailored financial services should be provided to meet material needs, and interventions with a focus on resilience and social support are particularly needed. Although licensed custodial grandparents were more likely to experience psychological distress due to their pre-existing vulnerability than unlicensed counterparts, parallel services should be provided to all kinship caregivers.

